# Overlap between irritable bowel syndrome and common gastrointestinal diagnoses: a retrospective cohort study of 29,553 outpatients in Germany

**DOI:** 10.1186/s12876-022-02118-y

**Published:** 2022-02-05

**Authors:** Sven H. Loosen, Karel Kostev, Markus S. Jördens, Tom Luedde, Christoph Roderburg

**Affiliations:** 1 Clinic for Gastroenterology, Hepatology and Infectious Diseases, Medical Faculty of Heinrich Heine University Düsseldorf, University Hospital Düsseldorf, Moorenstraße 5, 40225 Düsseldorf, Germany; 2 Epidemiology, IQVIA, Frankfurt, Germany

**Keywords:** IBS, Functional disorder, Gastrointestinal diseases, Symptoms, Diagnosis

## Abstract

**Background:**

Irritable bowel syndrome (IBS) represents the most common functional disorder of the gastrointestinal tract. Many patients with IBS display complex gastrointestinal (GI) symptoms leading to overlapping diagnosis of IBS and other GI diseases in many patients.

**Methods:**

Using the Disease Analyzer database (IQVIA) featuring patients treated within 2010 and 2019 within 1240 general practices in Germany, we analyzed the prevalence of common GI diseases within 12 months prior to and after the first diagnosis of IBS.

**Results:**

65,569 patients with an initial diagnosis of IBS were included into the analysis. Out of these, 29,553 patients had an observation time of at least 12 months prior to the first IBS diagnosis and at least 12 months after the first IBS diagnosis. Mean age was 48.8 (SD: 18.4) years, 65.0% were female. Notably, 16,164 (55%) of these patients had at least one preexisting diagnosis of another GI diseases within 12 months prior to the first IBS diagnosis. Most common overlapping diagnoses were intestinal infectious diseases (26%), gastritis/ duodenitis (21%), diseases of the esophagus (15%), non-infectious enteritis or colitis (7.4%), functional dyspepsia (6%) and ulcers (1.0%). Additionally, 12,048 (41%) received one of these diagnosis within 12 months after the first IBS diagnosis.

**Conclusion:**

Our data provide evidence for a high overlap between IBS and other GI diagnoses. Moreover, we show that IBS is frequently diagnosed in patients with preexisting GI diseases, potentially putting into question the validity of IBS diagnosis at least in some cases.

## Background

As early as 3000 years ago, Hippocrates described a patient with abdominal complaints, altered stool behavior, flatulence and urge to defecate [1]. In 1892, Osler and Hurst described a "mucous colitis" with discharge of mucus (mucorhea), cellular debris, and "intestinal sand" [2]. The term “spastic colon” or "irritable colon" was used by Ryle in 1928 as well as Jordan and Kiefer in 1929 [2], respectively, who described a musculo-neural disorder of the colon in 30% of gastroenterological outpatients with abdominal pain and impaired defecation. Various terms have since been used in the literature since then, however, the designation “irritable bowel syndrome” has become established in nowadays for the disease. According to the current S3 guideline [3], irritable bowel syndrome (IBS) disease is present when all 3 items are met: (1) there are chronic symptoms, i.e., lasting longer than 3 months (e.g., abdominal pain, flatulence), which are referred to the intestine by the patient and the physician and are usually accompanied by changes in bowel movements; (2) the complaints should justify the patient seeking help and/or worrying about them and be so severe that the quality of life is relevantly impaired as a result; (3) there are no characteristic alterations for other clinical pictures which are probably responsible for these symptoms. Nevertheless, in many cases suspected for an IBS, complaints are less specific and it is difficult to decide whether an IBS or another disease is responsible for the patients’ symptoms [3, 4]. It is therefore likely that the prevalence of IBS in Germany, reported at 1.34% in 2017 [5], is overestimated given the current definition of IBS. In the present study, we analyzed the proportion of patients with a diagnosis of IBS who, within one year prior to the diagnosis of IBS, received a diagnosis of another gastrointestinal disease that is in principle capable of causing symptoms that also fit IBS and might therefore have precluded the diagnosis of IBS.

## Methods

### Database

This study was based on data from the Disease Analyzer database (IQVIA), which contains drug prescriptions, diagnoses, and basic medical and demographic data obtained directly and in anonymous format from computer systems used in the practices of general practitioners and specialists [6]. The database covers approximately 3% of all outpatient practices in Germany. Diagnoses (according to International Classification of Diseases, 10th revision [ICD-10]), prescriptions (according to Anatomical Therapeutic Chemical [ATC] Classification system), and the quality of reported data are being monitored by IQVIA. In Germany, the sampling methods used to select physicians' practices are appropriate for obtaining a representative database of general and specialized practices. It has previously been shown that the panel of practices included in the Disease Analyzer database is representative of general and specialized practices in Germany [6]. Finally, this database has already been used in previous studies focusing on digestive system diseases [7–10].

### Study population

This retrospective cohort study included adult patients (≥ 18 years) with an initial diagnosis of irritable bowel syndrome (ICD-10: K58) from 1240 general practices in Germany between January 2010 and December 2018, who had an observation time of at least 12 months prior and a follow-up time of at least 12 months after the initial IBS diagnosis (index date).

### Study outcome

The outcome of the study was the prevalence of the following diagnoses noted as ‘confirmed’ within 12 months prior to or 12 months after the first IBS diagnosis: intestinal infectious diseases (ICD-10: A01-A09), gastritis/duodenitis (ICD-10: K29), esophagus diseases (ICD-10: K20-K22), functional dyspepsia (ICD-10: K30), ulcers (ICD-10: K25-K28), noninfective enteritis and colitis (IC-10: K50-K52), other diseases of stomach and duodenum (ICD-10: K31).

## Results

### Prevalence of preexisting GI diseases in patients diagnosed with IBS

**Fig. 1:**
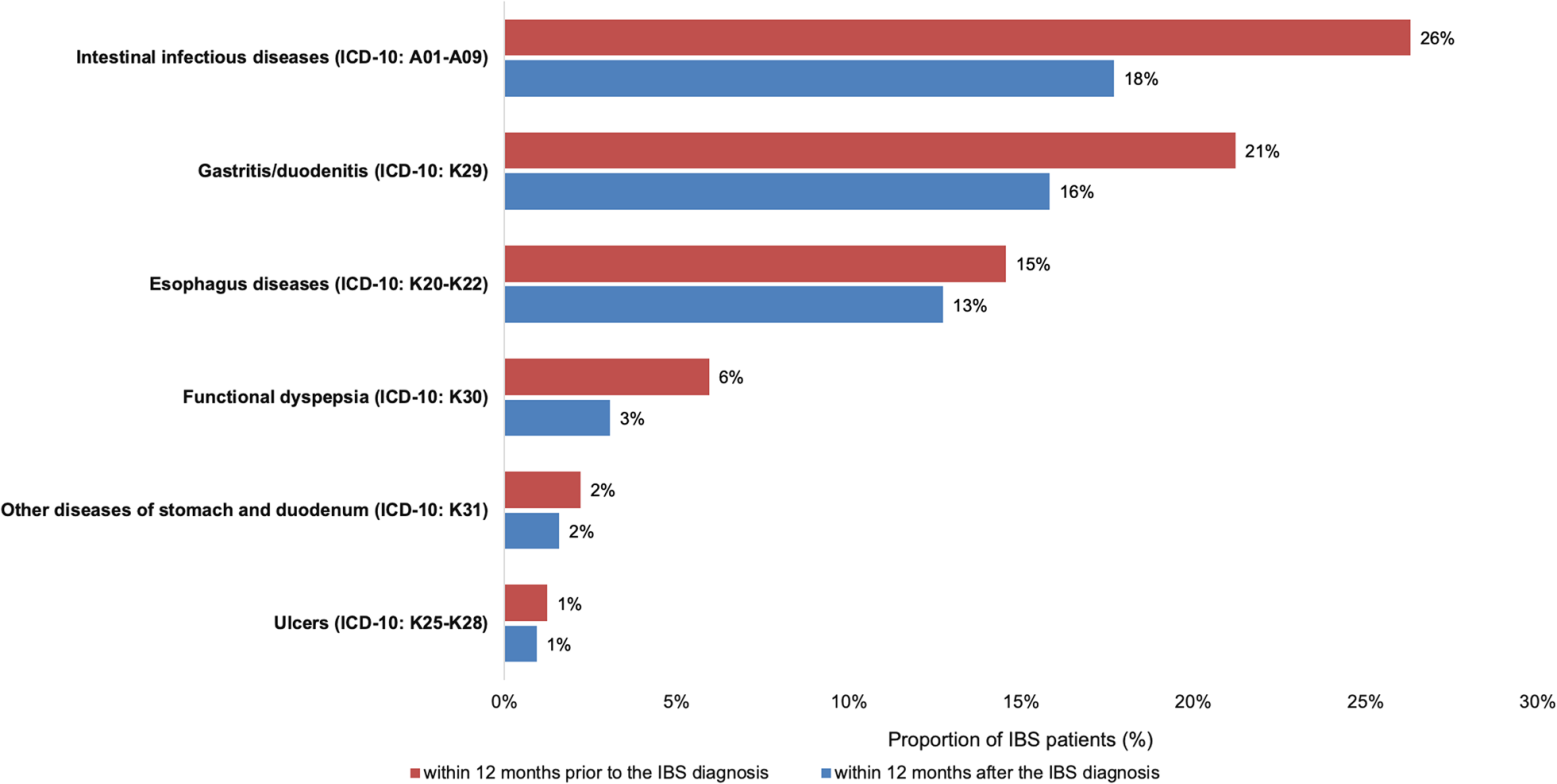
Proportion of patients diagnosed with IBS displaying at least one other GI-diagnosis twelve months before (red) or after (blue) the time-point of IBS diagnosis

Using the Disease Analyzer database (IQVIA) featuring patients treated between 2010 and 2019 within 1240 general practices in Germany, we analyzed the prevalence of established GI disorders like intestinal infectious diseases, gastritis/duodenitis, esophagus diseases, functional dyspepsia, ulcers, noninfective enteritis and colitis as well as other diseases of the stomach within 12 months prior to and 12 months after the first IBS diagnosis. Of 65,569 patients with an initial diagnosis of irritable bowel syndrome (IBS), 29,553 patients had an observation time of at least 12 months prior to and 12 months after the first IBS diagnosis. These patients were included into further analysis (baseline characteristics are summarized in Table 1). In this cohort, mean age was 48.8 (SD: 18.4) years, 65.0% were female. 16,164 (55%) of these patients had at least one diagnosis of one of the predefined GI diseases within 12 months prior to the first IBS diagnosis. Most common overlapping diagnoses were intestinal infectious diseases (26%), gastritis/duodenitis (21%), diseases of the esophagus (15%), non-infectious enteritis or colitis (6%), functional dyspepsia (2%) and ulcers (1%, Fig. 1).

**Table 1:** Basic characteristics of the study sample

Variable	Proportion affected among patients with IBS (%) n = 29,553
Age (Mean, SD)	48.8 (18.4)
Age 18–40	34.8
Age 41–50	20.1
Age 51–65	26.4
Age > 65	18.7
Women	67.2
Men	32.8

Proportions of patients in % given, unless otherwise indicated. *SD* standard deviation

### Incidence of other GI diseases after diagnosis of IBS

Further analyses revealed that 12,048 (41%) of patients received one of predefined GI diagnosis within 12 months following the first IBS diagnosis, further underpinning the overlap between IBS and other GI-diagnosis. In detail, these consisted of intestinal infectious diseases (18%), gastritis/ duodenitis (16%), diseases of the esophagus (13%), non-infectious enteritis or colitis (3%), functional dyspepsia (2%) and ulcers (1.0%, Fig. 1).

## Discussion

Irritable bowel syndrome (IBS) represents a common gastrointestinal (GI) functional disorder that affects the digestive system and causes symptoms such as stomach cramps, bloating, diarrhea and constipation. IBS can affect patients regardless of their age, sex, socioeconomic status or race, and, due to its chronic nature which significantly impairs quality of life, represents an enormous economic burden as IBS patients are more likely to require time off work an seek medical care [11]. As the diagnosis of IBS is a diagnosis of exclusion and typical IBS symptoms are also frequently observed in other GI disorders, the road to final diagnosis can be challenging and extensive in terms of time. Moreover, a recent study based on health insurance data from Germany suggest that patients with IBS are likely not receiving sufficient diagnostic evaluation in conformity with the relevant guidelines [5]. In the present manuscript, we aimed at determining the prevalence of an overlaps between IBS and other common GI disorders. We used the Disease Analyzer database (IQVIA), which contains diagnoses as well as basic medical and demographic data of over nine million outpatients in Germany, to identify GI diseases that were diagnosed within 12 months before and after diagnosis of IBS was made. In a cohort of 29,553 IBS patients, we show that there is an extensive overlap between the diagnosis of IBS and other GI disorders including intestinal infectious diseases, non-infectious enteritis and colitis, gastritis and duodenitis as well as disease of the esophagus. As such, 26% and 18% of IBS patients were diagnosed with an GI disease 12 month before or after the diagnosis of IBS, respectively.

Diagnosis of IBS is based on the presence of chronic abdominal symptoms as well as the exclusion of other organic diseases that might explain patients´ symptoms [3, 4, 12]. Main symptoms leading to the diagnosis of IBS include abdominal pain and changed bowel habits that are common to different other GI diseases. In many cases, it might therefore be challenging to clearly identify the specific etiology of patients´ complain and in particular the differentiating between IBS and other functional disorders such as functional dyspepsia [13] as well as organic gastrointestinal diagnoses including gastroesophageal reflux disease, erosive esophagitis or inflammatory bowel syndrome requires a high level of clinical experience [14–17]. In our cohort of IBS patients, we could show that 55% of patients had been diagnosed with a different GI disorder up to 12 months before the diagnosis of IBS was made. Likewise, 41% of patients were diagnoses with another GI disorder 12 months after diagnosis of IBS. Despite the fact that IBS can be diagnosed in the presence of other GI diseases, the German S3 guideline as well as international guidelines demand that there are no characteristic alterations for other clinical pictures which are probably responsible for these symptoms. Thus, our data call into question whether the diagnosis of IBS was made in all cases in accordance with current guidelines. In this line of thinking, our data put into question current data on the high prevalence of IBS in Germany and other Western countries. Nevertheless, it should be remembered that there are forms of IBS that specifically occur after viral or bacterial gastrointestinal infections and are then referred to as post-infectious irritable bowel syndrome (PI-IBS) [18, 19]. Finally, our data on an overlap of IBS and specific GI diseases underscore that IBS must not lead to a delayed diagnosis of other relevant organic disorders such as inflammatory bowel disease, chronic intestinal infectious disease, ulcers or even GI cancer, which require disease-specific therapies. In addition, we would like to point out that GI disorders, which were the focus of the current study, are not the only comorbidities of patients with IBS. A large number of studies have shown that a variety of extraintestinal comorbidities such as psychiatric disorders like anxiety, depression, somatization, chronic fatigue syndrome or sleep disturbance are highly relevant in the context of IBS as well [20–22].

Our study is limited by some methodological aspects that we would like to acknowledge. Diagnoses coded in the Disease Analyzer database are recorded based on the ICD-10 classification system that could be associated with a risk of undercoding or misclassification of diagnoses. Regarding IBS in particular, we are unable to provide information on the diagnostic steps performed by the physician to rule out other diagnoses prior to the diagnosis of IBS. Moreover, we acknowledge a lack of available data on lifestyle parameters and socioeconomic aspects including smoking status, alcohol use, physical activity, family status and employment, which are known to have an impact on the diagnosis of IBS [4]. Finally, our study did only include patients from the outpatient sector. Although, in our opinion, IBS is predominantly diagnosed in outpatient practices, the missing data on the hospital sector could lead to a selection bias.

## Conclusion

In summary, our data provide evidence that, on the one hand, the diagnosis of IBS in Germany is often preceded by different well-established GI disorders and, on the other hand, IBS patients are frequently diagnosed with other somatic GI diseases shortly after the diagnosis of IBS. This overlap between diagnoses could call into question the correct diagnosis of IBS, at least in some cases. Therefore, we believe that further research efforts including symptom-based subgroup analyses of IBS patients are warranted to further improve the diagnostic accuracy of IBS in future.

## Data Availability

The datasets used and analyzed during the current study are available from the corresponding author on reasonable request.

## Abbreviations

ATC: Anatomical therapeutic chemical
GI: Gastrointestinal
IBS: Irritable bowel syndrome
ICD-10: International classification of diseases, 10th revision
PI-IBS: Post-infectious irritable bowel syndrome
